# A prospective registry study of the epidemiology and management of childhood cancer in the Gambia—The first year experience

**DOI:** 10.1002/hsr2.70084

**Published:** 2024-09-23

**Authors:** Samuel Adegoke, Cherno Jallow, Olufunmilola Ogun, Wuday Camara, Musa Jaiteh, Peter Mendy, Gabriel Ogun, Ousman Leigh, Barry Pizer

**Affiliations:** ^1^ Department of Paediatrics Edward Francis Small Teaching Hospital (EFSTH) Banjul The Gambia; ^2^ Department of Paediatrics Obafemi Awolowo University Ile‐Ife Nigeria; ^3^ Department of Surgery, Paediatric Surgical Unit EFSTH Banjul The Gambia; ^4^ Department of Ophthalmology Sheikh Zayed Regional Eye Care Centre Kanifing The Gambia; ^5^ Department of Anatomic Pathology, Histopathology Unit EFSTH Banjul The Gambia; ^6^ Paediatric oncology Division University of Liverpool Liverpool UK

**Keywords:** challenges, epidemiology, outcome, pediatric cancers, successes, The Gambia

## Abstract

**Background and Aim:**

Globally, over 180,000 children develop cancers yearly, with about 80% residing in low‐ or middle‐income countries where cancer‐associated mortality is also high. In The Gambia, cumulative incidence rate of 27.6 childhood cancers/million population was reported between 2002 and 2011. The current study appraised newly‐established pediatric oncological services in The Gambia.

**Methods:**

In this prospective registry study, children with cancer who presented at the pediatric units, Edward Francis Small Teaching Hospital, Banjul, between November 2022 and October 2023 were assessed. Data on sociodemographic variables, mode of admission and presentation, tumor type, diagnostic methods, and challenges such as laboratory support, treatment, use of blood/blood products; and eventual outcome were analyzed.

**Results:**

The median (interquartile range, IQR) age at presentation of the 44 children was 36.0 (22.3–117.0) months. Wilms tumor was the most common tumor 12 (27.3%); followed by leukemia 11 (25.0%); germ cell tumor 8 (18.2%); lymphoma 6 (13.6%); retinoblastoma 4 (9.1%); rhabdomyosarcoma 2 (4.5%) and one central nervous system tumor (2.3%). The median(IQR) duration of symptoms before presentation was 48 (21–90) days, presentation to diagnosis 7.5 (3–20.8) days, and first symptom to diagnosis 62.5 (32–126.8) days. Treatment refusal and abandonment rates were 20.5% and 13.6%, respectively. Families of 93.8% of children could not procure cytotoxic drugs due to nonavailability, high cost, or both. Adequate laboratory monitoring was only available in 6.8%, and none had platelet concentrate transfusion or radiotherapy. The nine (20.5%) who completed treatment are currently being followed up, 10(22.7%) are still receiving chemotherapy, while 2(4.5%) were referred. Eight (18.2%) died, predominantly from metastasis (75%) and severe drug toxicities (25%).

**Conclusion:**

Late presentation and diagnosis, poverty, unavailability of drugs, suboptimal or lack of laboratory testing, blood product, adjuvant medications, and psychosocial supports contributed to high treatment refusal, abandonment, and mortality. These daunting challenges can be ameliorated with regular community sensitization, frequent cancer auditing, and strong political will.

## 1 INTRODUCTION

Childhood cancer, an important global public health problem, is a leading cause of pediatric disease‐related death globally. About 180,000 children develop cancers annually worldwide.^1^, ^2^ This figure could however be a significant underestimate, considering a 2019 model‐based study on global incidence of pediatric malignancy which estimated that 43% of children aged 0–14 years with malignancies were never diagnosed.^3^ In high‐income countries (HICs), about 140 children/million develop cancer annually, compared to about 70–100 children/million in low‐ and middle‐income countries (LMICs).^4^, ^5^ The highest burden is in low income countries (LICs) where majority of delayed or misdiagnosis occur and effective treatments are often unavailable, scarce, or unaffordable.^6^ The overall cancer 5‐year survival in HICs is about 80% (i.e., 110 of 140 children/million) but declines dramatically to about 30% or less in LMICs (20–30 out of 70–100 children/million).^1^ The incidence of and thus from childhood cancer in LMICs countries is likely to increase as deaths from malnutrition and infection decrease.

The incidence of cancer is roughly 20–30 times higher in adults compared to children. This translates to approximately 0.5%–4.6% of all newly diagnosed cancers based on data collected by population registries.^7^ Hospital‐based studies in sub‐Saharan Africa (SSA) estimate that pediatric cancers constitute about 1%–3% of all pediatric admissions.^8^ Based on data from National Cancer Registry in The Gambia, the age‐standardized incidence rate of cancer in children aged 0–14 years over a 10‐year period 1988–1998 was 34.7 per million population.^9^, ^10^ There was a 20% reduction of this value to 27.6 children per million population in the data obtained for the 10‐year period from 2002 to 2011.^11^ Effective pediatric oncological services are limited in most LICs. These include inadequate or absent cancer registries at the hospital, community, and national levels for effective collation of cancer cases; few or no specialized pediatric oncology centers with adequate patients' access, diagnostic facilities, or treatment; and limited number of experienced personnel including pediatric oncologists, nurses, surgeons, radiation therapists, and pathologists. Another significant challenge is relative or absolute unavailability of cytotoxic drugs and other resources for supportive care especially blood and blood products.^12^ Several other obstacles exist, such as patients delaying seeking medical attention, limited financial resources, and prematurely leaving treatment. These factors contribute to higher rates of complications and death from cancer. In these situations, the overall survival rate can be as low as 20% or less, compared to 80% observed in many developed countries.^13^

The current study was designed to appraise pediatric oncology services at the only teaching hospital in The Gambia. The pattern of childhood cancers, diagnosis and treatment challenges were examined. Practical suggestions for improving the diagnosis and management are highlighted.

## 2 METHOD

***Research Design*:** This is a registry study, including all children with cancer who presented at the pediatric medical or surgical units of Edward Francis Small Teaching Hospital (EFSTH), Banjul, The Gambia.

***Target Population:*** The study population included all children who presented and were diagnosed with cancer at EFSTH, The Gambia, between November 2022 and October 2023. The Gambia has a population of approximately two million people residing in five regions and one capital city, the Greater Banjul district. The regions are West coast Region, North Bank Region, Lower River Region, Central River Region, and Upper River region.

***Study setting:*** The EFSTH is the only tertiary hospital in the Gambia and hence, receives referrals from all over the country. It is a 500‐bed tertiary care institution and serves as the country's principal referral hospital. The Pediatric hematology–oncology unit was established in year 2022 and offer chemotherapy. As of 2023, there was only one pediatric hematologist–oncologist in the country.

### 2.1 Data collection

Data on the sociodemographic characteristics (age, sex, and socioeconomic class); medical history and physical findings were obtained for each patient through a standardized proforma. Relevant data on childhood cancer including age at diagnosis of cancer and age at first presentation at health facility were obtained. Other information obtained included mode of admission (direct admission from home or hospital referral; time of onset/duration of symptoms before presentation; interval between presentation to the hospital; and commencement of primary therapy). Others included methods of confirmation of diagnosis, interval between presentation at the hospital and confirmation of diagnosis; the type of tumors; and how it varies by age, sex, and parental socioeconomic class.

Data on therapy included type and timing of commencement of primary therapy; number who completed therapy; number who abandoned therapy; and reasons for discontinuation, number who refused therapy and reasons for refusal, for example, inability to afford treatment.

Challenges during therapy were assessed by obtaining information on availability of laboratory tests, cytotoxic drugs supportive therapies such as blood and blood product transfusion), antibiotics, antiviral, antifungal, antiparasitic agents, and use of granulocyte colony‐stimulating factor (G‐CSF).

Outcome measures included how many died, the likely cause(s) of death (metastasis or drug toxicity), referral and the survival rate.

### 2.2 Ethical consideration

Approval for the study was obtained from the Ethics and Research Committee of the EFSTH, Banjul, The Gambia (EFSTH_REC_2023_035. In addition, parents or caregivers of the children gave written consent to participate in the study after careful explanation of study objectives and its significance, right of participants to decline to participate or withdraw any time, and resolve to maintain confidentiality. Children older than 7 years also gave their assent before enrolling in the study.

### 2.3 Data analysis

Both descriptive and inferential statistics were employed to analyze the data using SPSS version 22. Frequencies and/percentages were used to summarize categorical variables, while median (interquartile range, IQR) were used for continuous variables. Categorical and continuous variables were compared between groups with the Chi‐squared test and Mann–Whitney U test, respectively, in a two‐sided test.

## 3 RESULTS

Forty‐five children presented to EFSTH with malignancy from November 1, 2022 to October 31, 2023. One of them who was previously treated for Hodgkin lymphoma in India was only on follow up care at our hospital, was not included in the subsequent analysis. The remaining 44 children formed the basis of our analysis.

***Sociodemographic characteristics*:** Age at presentation, gender, ethnic groups, and marriage type.

There were 26 (59.1%) males, with a male: female of 1.4:1. The median (IQR) age at presentation of the 44 children with malignancy was 36.0 (22.3–117.0) months. The youngest child was 10 days, while the oldest was 168 months, that is, 14 years. The majority, 26 (59.1%) were under five, followed by adolescents (≥10 years), 10 (22.7%), and the least group was aged 5–9 years, with eight (18.2%) children.

Among the patients, the Fula ethnic group was the most prevalent at 40.9% (18 patients). Mandinka followed at 34.1% (15 patients), then Wolof at 11.4% (five patients), and Jola at 4.5% (two patients). The remaining 9.1% (four patients) belonged to other ethnicities. Three of these patients were from The Gambia (Serrahuleh, Manjago, and Serrel, one each) and one patient belonged to the Fanti tribe in Ghana.

Twenty (45.5%) of the children were from consanguineous marriages, with 13 (65.0%) being Fula, four (20.0%) Mandinka, and three (15.0%) Wolof.

### 3.1 Types of cancer

Wilms tumor (WT), 12 (27.3%) was the most common tumor identified, followed by leukemia, 11 (25.0%); germ cell tumor (GCT) eight (18.2%); lymphoma six (13.6%); retinoblastoma four (9.1%), rhabdomyosarcoma two (4.5%), and central nervous system (CNS) tumor one (2.3%).

***WT:*** Ten (83.3%) of the 12 children had localized tumor and two (16.7%) metastatic tumor (lungs and liver). The right kidney was affected in seven (58.3%) children, left kidney in four (33.3%) children while one (8.3%) had bilateral WT.

***Leukemia**:* Nine (81.8%) of the 11 children had Acute Lymphoblastic Leukemia, while two (18.2%) had Acute Myeloblastic Leukemia (AML). None had chronic leukemia.

***GCT:*** Three (37.5%) of the eight children had mature ovarian teratoma, two (25.0%) had mature testicular teratoma, another two (25.0%) had yolk sac (endodermal sinus) tumor, and one (12.5%) had sacrococcygeal teratoma.

***Lymphoma:*** Three (50.0%) of the six children had Hodgkin's lymphoma, two (33.3%) had Burkitt lymphoma, and one (16.7%) had non‐Hodgkin non‐Burkitt lymphoma.

***Retinoblastoma:*** Two (50.0%) of the four children had bilateral eye involvement, while one each (25.0%) had unilateral involvement.

### 3.2 Relationship between cancer types and sociodemographic variables (age, sex, and ethnicity)

#### 3.2.1 Types of cancer and age distribution

According to Table 1, which details the age distribution of the 44 children diagnosed with cancer, WT, retinoblastoma, and GCT were most frequently diagnosed in very young children, specifically those under 5 years old. Among children with WT, a high proportion (83.3%, or 10 out of 12) were diagnosed before the age of five. Similarly, a substantial percentage of children with GCT (87.5%, or seven out of eight) and all four children with retinoblastoma were diagnosed in this young age group. All cases of lymphoma were seen in children 5 years or above, and seven (63.6%) of 11 cases of leukemia affected those older than 5 years. About a third, four (36.4%) of the children with leukemia were younger than 5 years of age.

**Table 1:** Age group distribution, median, lowest and highest age at presentation of the 44 children with cancer.

Cancer type	Age group 0–4 (years)	5–9 (years)	10–14 (years)	Median age at time of presentation (months)	Lowest age at time of presentation (months)	Highest age at presentation (months)
**Wilms tumor** *N* (%) = 12 (27.3)	10	1	1	22.5	0.3	132.0
**Lymphoma** *N* (%) = 6 (13.6)	0	3	3	120.0	72.0	168.0
**Leukemia** *N* (%) = 11 (25.0)	4	4	3	96.0	8.0	144.0
**Rhabdomyosarcoma** *N* (%) = 2 (4.5)	1	0	1	84.0	48.0	120.0
**Central nervous system tumor** *N* (%) = 1 (2.3)	0	0	1	120.0	120.0	120.0
**Germ cell tumor** *N* (%) = 8(18.2)	7	0	1	24.0	6.0	144.0
**Retinoblastoma** *N* (%) = 4 (9.1)	4	0	0	36.0	2.0	41.0
**Total** *N* (%) = 44 (100.0)	26 (59.1)	8 (18.2)	10 (22.7)	36.0	0.3	168.0

The median age of the children at presentation for WT, GCT, and retinoblastoma were 22.5 months, 24 and 36 months, respectively. For lymphoma and CNS tumor, the median age of the patients at presentation was 120 months (i.e., 10 years). Table 1 also shows the types of cancer and median age at presentation

#### 3.2.2 Types of cancer and gender

**Figure 1:**
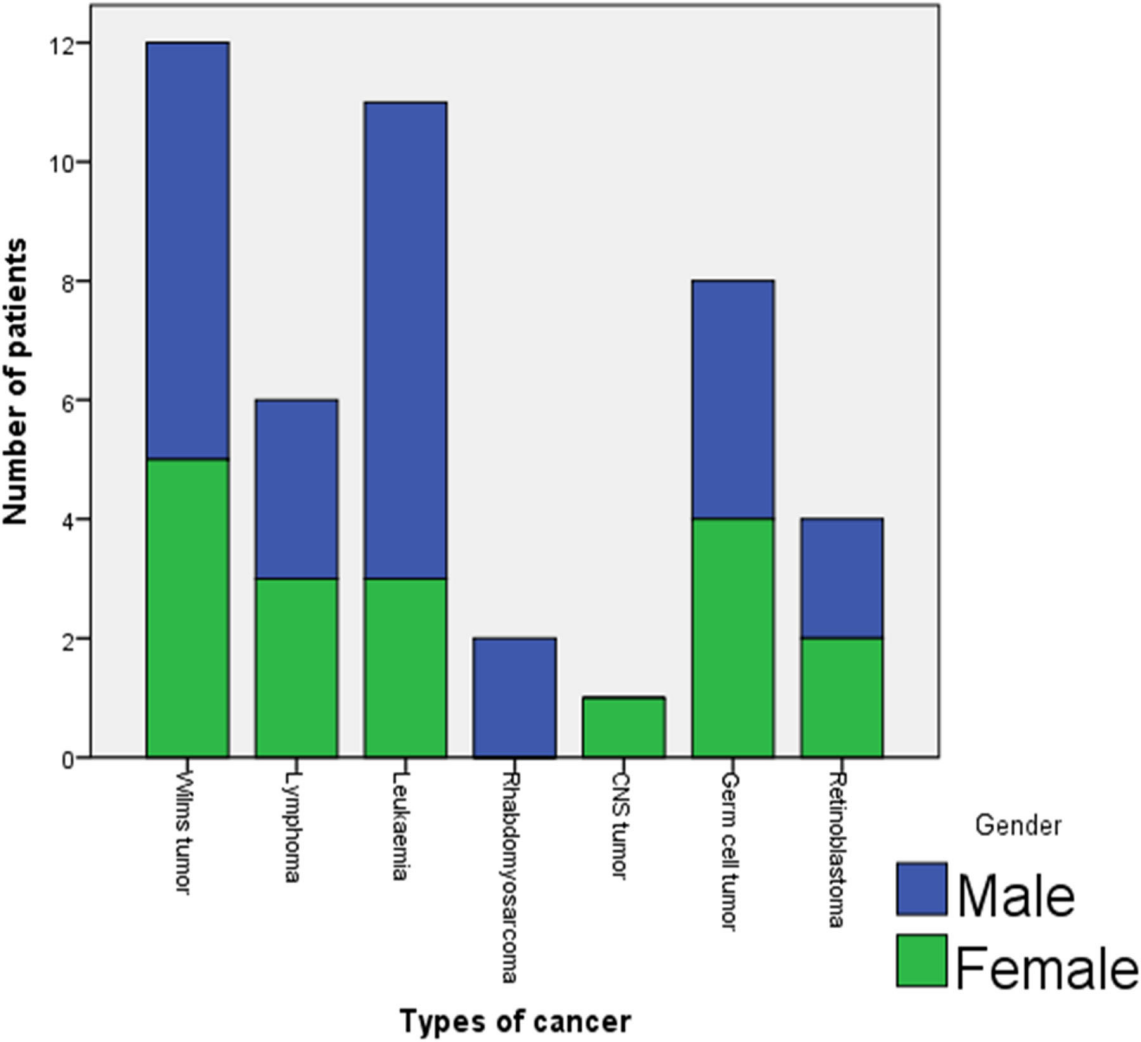
Gender distribution of the 44 children with malignancy.

Figure 1 shows the gender distribution. Almost equal proportions of male and females were affected in all the cancer types, except in leukemia where there was a distinct male preponderance (72.7% males vs. 27.3% females), although the difference was not statistically significant, *p* = 0.479.

#### 3.2.3 Types of cancer and ethnicity

Seven (38.9%) of the 18 Fula children compared to five (19.2%) of the 26 non‐Fula children had WT. Although, a higher proportion of Fula children had WT, the difference was not statistically significant, *χ* ^2^ = 2.072, *p* = 0.150. However, a significantly higher proportion of Fula children, four (22.2%) of 18, as against none of the non‐Fula had retinoblastoma, *χ* ^2^ = 3.951, *p* = 0.047 (Table S1).

#### 3.2.4 Types of cancers and consanguinity

**Figure 2:**
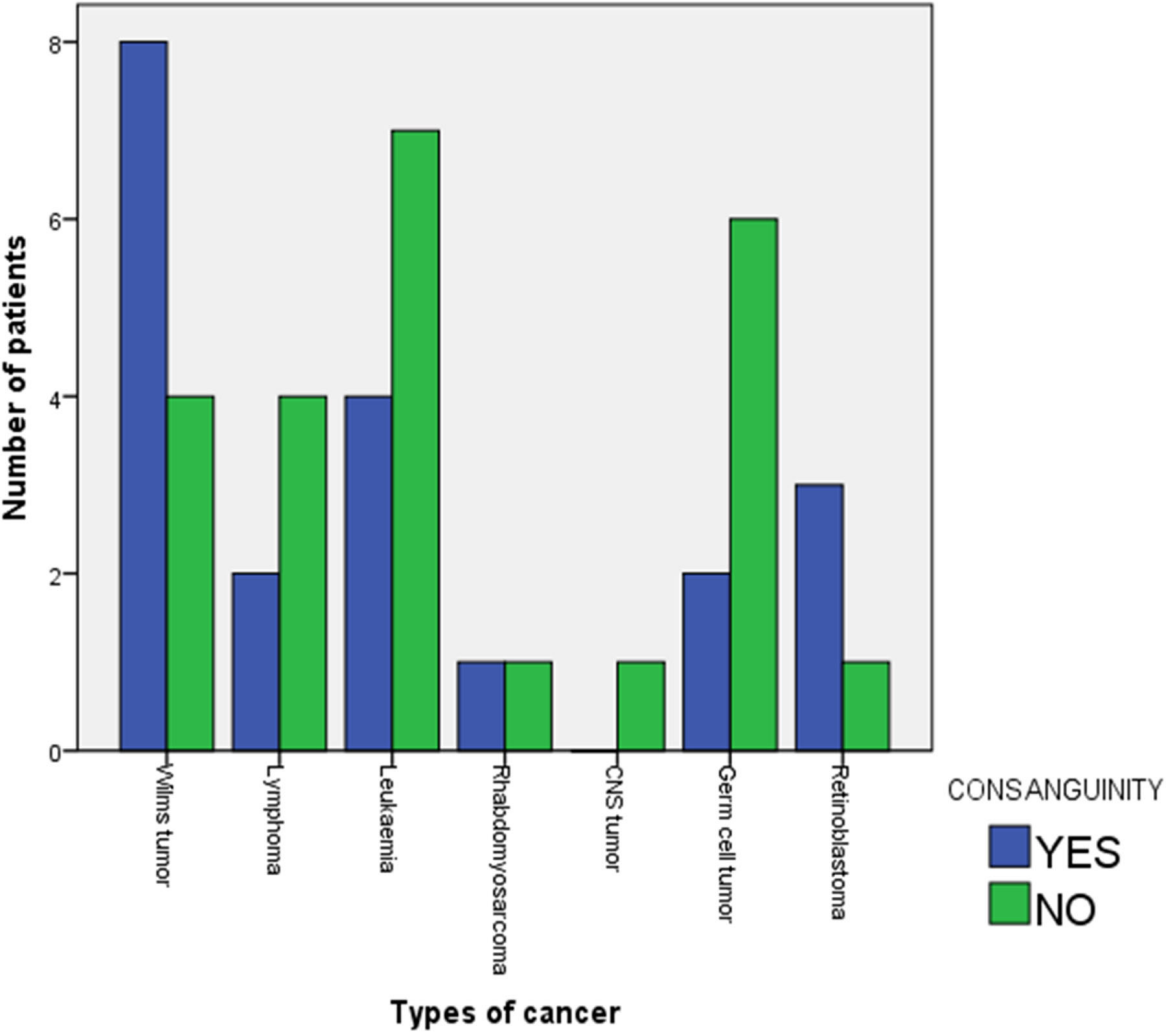
Relationship between types of cancers and consanguinity.

As illustrated in Figure 2, WT and retinoblastoma appeared more frequently in children born to consanguineous parents. Specifically, eight out of 12 children (66.7%) with WT and three out of four children (75.0%) with retinoblastoma had parents who were blood relatives.

### 3.3 Mode of presentation

The principal complaints at presentation were swelling or mass, seen in 27 (61.4%), acute illness such as fever, pallor, difficulty in breathing, seen in 12 (27.3%), bleeding in four (9.1%), and weight loss in five (11.4%). Some of the patients had more than one major complaint at presentation.

### 3.4 Interval between onset of symptoms and presentation in our facility, presentation and diagnosis, and lag time between first symptom at home and eventual diagnosis in our unit

The median (IQR) duration of symptoms before presentation was 48 (21–90) days, ranging from three to 240 days. From presentation to diagnosis, it took a median (IQR) of 7.5 (3–20.8) days, with minimum and maximum interval of 2 and 178 days, respectively. On the whole, the median (IQR) lag time (period between onset of first symptom at home to eventual diagnosis in our hospital) was 62.5 (32–126.8) days, ranging from 5 to 358 days.

The median duration of symptoms before presentation among the 20 who were admitted directly from home (33 days) was similar to the 12, who were referred from other hospitals (30 days), Mann–Whitney‐U (Z test) = 0.115, *p* = 0.737.

### 3.5 Median duration of symptoms, duration of presentation to diagnosis and lag time (first symptom to diagnosis), and types of cancer

Table 2 shows the median duration of symptoms, duration of presentation to diagnosis and lag time (first symptom to diagnosis), and types of cancer. While patients with WT, lymphoma, leukemia, and retinoblastoma presented within 30 and 60 days (1–2 months) of onset of symptoms, those with rhabdomyosarcoma and GCT presented later within 90 and 135 days. Diagnosis was fastest in those with retinoblastoma (2 days) and longest in those with rhabdomyosarcoma (30 days). The median time taken to make diagnosis from onset of symptom was almost 2 months in WT (51.5 days), leukemia (52 days), and retinoblastoma (62 days). This lag time was longest in children with rhabdomyosarcoma (120 days) and GCT (139.5 days).

**Table 2:** Median duration of symptoms, duration of presentation to diagnosis and lag time (first symptom to diagnosis), and types of cancer.

Types of cancer	Median duration of symptoms before presentation (days)	Median duration between presentation and diagnosis (days)	Lag time (first symptom to eventual diagnosis in our unit (days)
Wilms tumor	45.0	6.0	51.5
Lymphoma	40.5	5.5	81.5
Leukemia	30.0	20.0	52.0
Rhabdomyosarcoma	90.0	30.0	120.0
Germ cell tumor	135.0	10.0	139.5
Retinoblastoma	60.0	2.0	62.0
**Total**	**48.0**	**7.5**	**62.5**

### 3.6 Modality of diagnosis

Ultrasonography helped diagnose malignancy in 12 (27.3%) of the cases, alongside clinical suspicion. These included eight with WT and four with retinoblastoma. Peripheral blood film and or bone marrow studies were used to make diagnosis in the 11 children with leukemia. For other solid tumors, cytology of fine needle aspirate was used in five (11.4%), and for the remaining 20 (45.5%), histology of tissue biopsy was done to confirm diagnosis.

### 3.7 Interval between presentation and commencement of standard therapy

For those who had chemotherapy or surgery, the median (IQR) interval between presentation and commencement of standard therapy was 14.5 (4.0–42.0) days. The shortest interval was 2 days while the longest was 241 days. The median interval between presentation and initiation of treatment was short in retinoblastoma (2 days) and WT (11 days), but long in lymphoma (60.5 days), GCT (65 days) and rhabdomyosarcoma (74 days). Table 3 below shows interval between presentation and commencement of primary therapy.

**Table 3:** Interval between presentation and commencement of primary therapy.

Cancer type	Frequency	Median (days)	Minimum (days)	Maximum (days)
Wilms tumor	10	11.0	3	88
Lymphoma	2	60.5	13	108
Leukemia	10	24.5	2	45
Rhabdomyosarcoma	2	74.0	31	117
Central nervous system tumor	1	14.0	14	14
Germ cell tumor	8	65.0	2	241
Retinoblastoma	3	2.0	2	2
**Total**	**36**	**14.50**	**2**	**241**

### 3.8 Treatment modalities at presentation

Seven in 10 (72.7% or 32 patients) received chemotherapy. The remaining patients (12 or 27.3%) required different treatment approaches. Six patients with GCT containing mature teratomas underwent surgery only. Five patients who could not tolerate chemotherapy due to their unstable health condition received palliative care. One 10‐day‐old infant with WT had surgery.

### 3.9 Rate of refusal and abandonment of therapy

Treatment was refused by caregivers for nine (20.5%) patients. These children had the following diagnoses: four with lymphoma (including two cases of Burkitt lymphoma and two Hodgkin lymphoma), three with leukemia, one with rhabdomyosarcoma, and one with a GCT.

A total of six (13.6%) parents/caregivers discontinued chemotherapy treatment. Among them, four stopped treatment after receiving only one or two rounds, while the other two discontinued postsurgical chemotherapies. Notably, four out of these six patients had WT, while the remaining two had lymphoma and GCT, respectively.

### 3.10 Reasons for treatment refusal or abandonment

Out of the 15 parents/caregivers who refused or discontinued treatment, the main reason for most (seven, representing 46.7%) was the high cost of care. Another six (40.0%) opted for alternative, likely traditional, treatment methods, while the remaining two (13.3%) cited hopelessness about the patients' survival.

Thirty (93.8%) of the 32 patients who needed chemotherapy could not procure cytotoxic drugs. Only two (6.2%) were able to procure drugs without delay. The principal reasons given for not procuring were nonavailability of the drugs in the hospital pharmacy or in the country, 11 (36.7%); nonaffordability of drugs, five (16.7%), or both, 14 (46.7%).

Adequate laboratory monitoring was only available in three (6.8%) patients. The majority relied entirely on the full blood count services provided free of charge at the pediatric laboratory.

Only whole blood transfusion was available in the hospital. None of the eight patients who needed platelet concentrate transfusion received it.

Only one (5.9%) of the 17 patients who developed severe leucopaenia with or without febrile neutropaenia received G‐CSF, because of nonavailability.

None of the 44 patients had formal psychosocial support.

### 3.11 Final outcome

The survival outcome could not be ascertained in 17 (38.6%) of the 44 patients, comprising of nine (20.5%) who refused treatment, six (13.6%) who abandoned treatment, and two (4.5%) who were referred. As at October 30, 2023, nine (20.5%) of the 44 patients had completed their treatment protocol with evidence of remission and are currently being followed up. They included three (25.0%) of the 12 with WT and six (75.0%) of the eight with GCT. Another 10 (22.7%) patients were still receiving chemotherapy mostly on outpatient basis. They included six (54.5%) of 11 with leukemia, two (16.7%) of 12 with WT, one (25.0%) of the four with retinoblastoma and the child with CNS tumor. Three children with leukemia were in the maintenance phase of treatment.

Eight (18.2%) of the 44 children died.

### 3.12 Causes of death

The eight that died included three (25.0%) of the 12 with WT, two (18.2%) of 11 with leukemia and one each among those with lymphoma (16.7%), rhabdomyosarcoma (50.0%), and retinoblastoma (25.0%). Six (75.0%) of the eight died of metastasis (respiratory, renal and liver failure); one child died of severe febrile neutropaenia and one who had AML in addition to having Down syndrome and congenital heart disease died of severe pneumonia, heart failure, and pancytopaenia.

## 4 DISCUSSION

This study highlighted patterns of presentation, challenges, and successes associated with childhood cancers in the only university teaching hospital in The Gambia, in the first year after establishing a pediatric oncology unit. The study also discussed the prospects and strategies needed to improve pediatric cancer services in the country. In The Gambia, pediatric cancers receive little attention from both the national policymakers and global health agencies because of paucity of comprehensive national data on childhood cancer.

The median age at presentation of the children in this study was 36.0 months, with more than half being children under 5 years. Patients in this study were younger compared to children with cancers who presented at median age of 7 years at Ugandan Cancer Institute and 10 years for those at Kampala Cancer Registry.^14^ Possibly because more of the patients in our study had WT which were more common in those aged 1–4 years.

The finding of more males in this study is similar to findings in studies in the United States, Northern and Southern parts of Nigeria.^15^, ^16^, ^17^ The exact mechanisms for the gender differences in childhood cancer remains largely unknown.

The present study examined the influence of consanguinity on the development of cancer. Although, no significant relationship was found between endogamous marriage and carcinogenesis, a higher proportion of Fula children from consanguineous marriages had retinoblastoma and WT. In the literature, only one case of WT associated with consanguinity was found, a 4‐year old female whose parents were first‐degree relatives.^18^ Both retinoblastoma and WT are associated with deletion of an arm of chromosome, perhaps consanguinity influences this deletion.

Findings on the link between consanguinity and carcinogenesis in children are inconsistent, less predictable and needs further exploration. Among highly endogamous Qataris population, consanguinity had no effect on the overall incidence of cancer. Although, increase risk of leukemia, lymphoma, colorectal cancer, and lower risk of female cancers were documented.^19^ In United Arab Emirate where the rate of consanguineous marriage is about 50.5%, there appeared to be a causal link between childhood leukemia and consanguinity, but not with lymphoma.^20^, ^21^ It has been proposed that consanguinity creates a genetic predisposition to develop a toxin‐induced disease,^22^ and may increase frequency of high‐risk genotypes for enzymes that cause accumulation of xenobiotic, thereby explaining the higher consanguinity rate associated with leukemia.^22^

WT was the leading tumor, followed by leukemia, GCT, lymphoma, retinoblastoma, rhabdomyosarcoma, and CNS tumor. In a large study involving 21 centers in SSA on the pattern of cancer distribution, WT was found to be the most common solid tumor in Africa, with relative frequency of up to 20% of all pediatric cancers in some countries.^23^

Delayed presentation was a major challenge to effective cancer management in this study. Although this study did not find out reasons for the delayed presentation, a previous systematic review showed low health literacy as a major barrier.^24^ Others included lack of finances to meet treatment and transport costs, misdiagnosis, cultural belief that cancer is a product of witchcraft, role of faith healing, and strong belief in alternative medical care for cancer.^25^

The experience of delayed presentation is similar to many other cancer centers in Africa, where it is estimated that 50%–80% of patients with pediatric cancer in SSA present at an advanced stage.^26^, ^27^ Delay could occur at any time between symptom onset to treatment initiation. In addition to patients' delay seeking care, a study at a regional cancer center in Tanzania showed that referral delay or visit to traditional homes was a major component of treatment delay in childhood cancer management.^26^ Increase cancer awareness, training, and retraining of general medical officers in the Provinces, and strengthening of the referral process from lower‐level to higher‐level facilities will help reduce delay referral.

Also, diagnosis was further delayed after presentation. Previous review has shown that delayed diagnosis of pediatric cancer arises from fewer number of trained pathologists and specialized oncological centers.^28^ In The Gambia, there is only one Consultant Pathologist in the country at present, as against recommended one pathologist serving about 20,000 people in HICs. In many other counties in Africa, 0.5–1 million people are served by only one pathologist.^29^, ^30^, ^31^ Another major challenge in diagnosis was the unavailability of immunohistochemistry, molecular and genetic analyses, especially for patients with leukemia.

Comparable to many other findings in SSA, there was additional delay in commencing treatment after diagnosis. The principal reason for this delay was inability to procure cytotoxic drugs when needed (93.8%) either because of high cost, or nonavailability in the country or both. Gambia is a low income country with Gross Domestic Product of about 2.27 Billion USD and GDP per capital of 840 USD, with 53.4% of the population living below global poverty index of USD 1.25 per day.^32^, ^33^

Adequate basic hematologic and biochemical laboratory monitoring were only available in 6.8% of the patients. Cytogenetics and other molecular assessments were not available. Blood support or the use of supportive drugs like granulocyte colony‐stimulating factor and erythropoietin were not commonly available. Notably none of the patients that needed platelet concentrate transfusion had it.

Also, none of our patients had radiotherapy. This mirrors what is obtainable in most countries in SSA where only one in about five cases (18%) of radiotherapy need is met.^34^, ^35^ None of the patients had organized medical or psychosocial support when needed. Good cancer support system should include good infection control, nutritional support, family and spiritual/religious support.

Treatment refusal and abandonment rate was 34.1% (20.5% and 13.6%, respectively), principally because of inability to afford care. Treatment refusal or abandonment is multifactorial and is influenced by both social and disease‐specific factors, majorly financial problems (30%), unwillingness to lose any part of the body, for example, the eye in cases of enucleation (20%), long distance to treatment centers and preference for alternative medicine.^36^, ^37^ Treatment refusal or abandonment has been shown to worsen disease outcomes and may partly explain the survival disparity between cancer patients living in LICs and HICs.^38^

Although <20% of the children died at the end of the 1‐year study period, it is difficult to draw any conclusion regarding survival or mortality, since more than 40% of the children were still receiving treatment at the time and 34.1% had either refused or abandoned treatment. In many reports from SSA, survival rate of children with cancer is still low. Three‐quarter of the children who died had evidence of metastasis, indicating late presentation, a major contributor to mortality in many reports.^14^

This study has some limitations. One, the study was a single‐center with a small study population. Second, in‐depth individual interviews or focused group discussion of healthcare providers could have provided further insight into the causes of treatment refusal and abandonment, rather than relying on the caregivers alone.

The current number of children diagnosed to have cancer in the country is a tip of iceberg. Many children remain undiagnosed (either they do not present, or are misdiagnosed), and those that present are faced with lots of challenges. These daunting challenges can be ameliorated with regular community sensitization, frequent cancer auditing, and strong political will. To improve oncological services in the country, sustained collaboration with international organizations and strong advocacy are needed.^39^ Pediatric cancer unit must be fully supported, cancer care specialists (nurses, clinicians, pathologists, radiologists, surgeons, and other support staff) must be regularly trained and retrained; standardized treatment protocols should be advocated, and needed drugs/supports must be regularly provided. As brilliantly captured by Molyneux et al.,^28^ “rather than finding these hurdles disheartening, we will take them as personal challenges, and steadfastly continue to provide care until we succeed.”

## AUTHOR CONTRIBUTIONS

**Samuel Adegoke**: Conceptualization; methodology; data curation; investigation; writing—original draft; writing—review and editing. **Cherno Jallow**: Conceptualization; methodology; writing—review and editing. **Olufunmilola Ogun**: Conceptualization; methodology; writing—review and editing. **Wuday Camara**: Conceptualization; methodology; writing—review and editing. **Musa Jaiteh**: Conceptualization; methodology; writing—review and editing. **Peter Mendy**: Conceptualization; methodology; writing—review and editing. **Gabriel Ogun**: Conceptualization; methodology; writing—review and editing. **Ousman Leigh**: Conceptualization; methodology; writing—review and editing. **Barry Pizer**: Conceptualization; methodology; supervision; writing—review and editing.

## CONFLICT OF INTEREST STATEMENT

The authors declare no conflict of interest.

## TRANSPARENCY STATEMENT

The lead author Samuel Adegoke affirms that this manuscript is an honest, accurate, and transparent account of the study being reported; that no important aspects of the study have been omitted; and that any discrepancies from the study as planned (and, if relevant, registered) have been explained.

## Supporting information

Supporting information.

## Data Availability

The data that support the findings of this study are available on request from the corresponding author (S. A. A.).
