# Supplementary material for: A prospective registry study of the epidemiology and management of childhood cancer in the Gambia—The first year experience
Source: Health Sci Rep. 2024 Sep 23;7(9):e70084. doi: 10.1002/hsr2.70084 (PMC11420287; doi:10.1002/hsr2.70084)
Supplement: Supplementary file 1 — Supporting information. [file HSR2-7-e70084-s001.docx]

**Relationship between types of cancer and ethnicity**

| **Cancer type** | | **Tribes** | | | | | **Total** |
| --- | --- | --- | --- | --- | --- | --- | --- |
|  |  | **Fula** | **Mandinka** | **Wolof** | **Jola** | **Others*** |  |
|  | Wilms tumor | 7 | 3 | 1 | 0 | 1 | 12 (27.3) |
|  | Lymphoma | 2 | 1 | 3 | 0 | 0 | 6 (13.6) |
|  | Leukaemia | 2 | 6 | 0 | 1 | 2 | 11 (25.0) |
|  | Rhabdomyosarcoma | 1 | 1 | 0 | 0 | 0 | 2 (4.5) |
|  | CNS tumor | 0 | 0 | 0 | 0 | 1 | 1 (2.3) |
|  | Germ cell tumor | 2 | 4 | 1 | 1 | 0 | 8 (18.2) |
|  | Retinoblastoma | 4 | 0 | 0 | 0 | 0 | 4 (9.1) |
| **Total** | | **18 (40.9)** | **15 (34.1)** | **5 (11.4)** | **2 (4.5)** | **4 (9.1)** | **44 (100.0)** |

*Others: One child each from Serrahuleh, Manjago, Serrel in The Gambia, and one child from Fanti tribe in Ghana.
